# Data for indirect load case estimation of ice-induced moments from shaft line torque measurements

**DOI:** 10.1016/j.dib.2018.05.115

**Published:** 2018-05-28

**Authors:** R.J.O. de Waal, A. Bekker, P.S. Heyns

**Affiliations:** a Sound and Vibration Research Group, Department of Mechanical and Mechatronic Engineering, Stellenbosch University, Stellenbosch, South Africa; b Centre for Asset Integrity Management, University of Pretoria, Pretoria, South Africa

## Abstract

During ice navigation, blade measurements of ice-induced moments on ship propellers, are challenged by the harsh operating environment. To overcome this problem, shaft line measurements are performed inboard, and the required propeller loads are subsequently estimated using a dynamic model and the solution of an inverse problem. The inverse problem is mathematically ill-posed and requires the determination of the ice-induced moment on the propeller blades from shaft line measurements. Full-scale torsional response data is presented as calculated from indirect strain measurements on the shaft line of a polar supply and research vessel. The vessel operated on a 68-day voyage between Cape Town and Antarctica and spent almost 11 days in sea ice with observed concentrations above 90% and a maximum thickness of 3 m. Data for five ice-induced load cases are presented, including the shaft torque from indirect measurements and the estimated ice-induced moment, which is obtained by solving an ill-posed inverse problem. The ice-induced moments on the propeller are obtained by approximating the drive-train as a viscously damped, elastic lumped mass model. The ice-induced moment is then determined through existing approaches to solving the ill-conditioned inverse problem. The lumped mass model is presented along with algorithms to solve the inverse problem, including truncated singular value decomposition, truncated generalized singular value decomposition and Tikhonov׳s method. The resulting time series data for the inversely calculated ice-induced moments is published to provide industry with load cases for ice-going propulsion design.

**Specifications Table**

**Table:** 

Subject area	*Engineering*
More specific subject area	*Polar shipping*
Type of data	*Table, text file*
How data was acquired	*Strain gauge measurements through a LORD MicroStrain V-Link LXRS Wireless 7 channel analog sensor node and a WSDA-Base.*
Data format	*Raw and processed*
	*Example:*
	***Case1_ShaftTorque.txt***
	• *Time – Time vector* • *ShaftTorq_Normal – Torque calculated from strain measurements* • *ShaftTorq_ExHydro – Processed: Torque calculated from strain measurements – hydrodynamic torque. This implies that a constant motor torque is applied to overcome hydrodynamic resistance.*
	***Case1_IceTorque.txt***
	• *Time – Time vector* • *IceInd_Torq_TSVD – Processed: Inversely determined ice-induced propeller torque using Truncated Singular Value Decomposition.* • *IceInd_Torq_TGSVD – Processed: Inversely determined ice-induced propeller torque using Truncated Generalized Singular Value Decomposition.* • *IceInd_Torq_Tikh – Processed: Inversely determined ice-induced propeller torque using the Tikhonov method.*
Experimental factors	*Data was captured using a Höttinger Baldwin Messtechnik (HBM) Quantum, which received data from a V-link system and converted the digital signal to strain using a scale obtained from calibrating the node, and passed the data through an aliasing filter.*
Experimental features	*The response of ice-induced loading on the shaft line of a polar supply and research vessel was recorded using strain gauges. This data was processed through inverse methods in order to determine the ice-induced moment on the propeller of the vessel.*
Data source location	*SA Agulhas II Polar Supply and Research Vessel, during her 2015/2016 relief voyage between Cape Town and Antarctica.*
Data accessibility	*Data is provided with this article.*
Related research article	[19]*‘Indirect load case estimation for propeller-ice moments from shaft line torque measurements’, Cold Regions Science and Technology, 151, pp.237-248,*doi:10.1016/j.coldregions.2018.03.016.

**Value of the Data**

• Shaft-line torque data are presented for operational incidences of propeller ice impacts on a polar supply and research vessel on a voyage between Cape Town and Antarctica. The data included the maximum ice-impacts as measured on the shaft-line, although this does not necessarily imply that these load cases are also the maximum ice-induced moments on the propeller blade.
• Time histories of the induced propeller torque are published as determined through inverse moment calculations, by using a viscously damped, elastic, lumped mass structural model.
• This data provides industry with operational load cases for ice-going propulsion design.
• The co-publication of time histories from inversely calculated shaft moments enables the validation and further development of methodologies for inverse moment estimation.

## 1 Data

To determine the loading contribution of ice impact, the hydrodynamic torque was subtracted from the measured internal torque and the direction of the moment inverted to obtain a positive external ice-induced moment on the propeller. It was further evaluated if the estimated ice moment could again be inverted to match the measured shaft torque value. This inverted internal torque was obtained by determining the relevant external moment through the Tikhonov method and using this result as an input to the dynamic model to obtain the internal torque (by solving the forward problem).

**Fig. 1:**
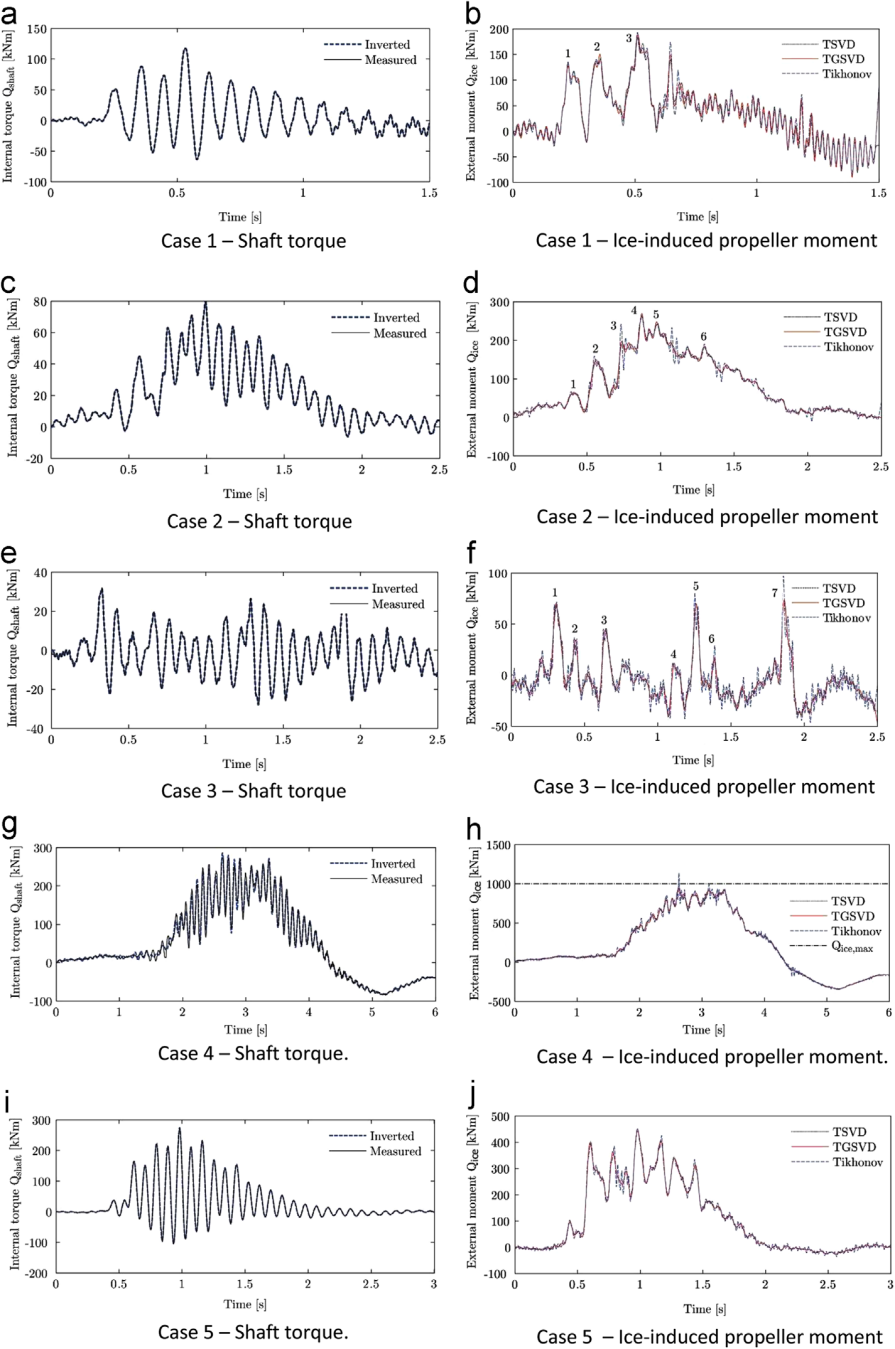
Time histories for five load case data sets containing indirectly measured shaft torque and inversely calculated ice-induced propeller moments.

The time series data of five propeller ice impact cases are published here as shown in Fig. 1. The ice impacts were identified through indirect measurements on the port-side shaft line of a polar supply and research vessel during ice passage in Antarctica. The operational conditions of the vessel are summarized in Table 1.

**Table 1:** Operating conditions during five propeller-ice impact conditions. Case 1, 2 and 5 were extracted from data on 12 December, Case 3 on 13 December 2015 and Case 4, on 11 December 2015. Average values of machine control and data for the ice contact duration and hydrodynamic torque, Q_h_, at the start of the ice contact condition are included.

Case	Start Time	Speed	*Q*_*h*_	Motor speed	Motor power	Propeller pitch	Average ice concentration	Ice thickness	Floe size
	[hh:mm:ss]	[knots]	[kNm]	[rpm]	[kW]	[%]	[%]	[cm]	[m]
Case 1	09:27:16	5.0	219.2	109.0	2270.0	88	2	110	60
Case 2	09:52:52	5.4	145.0	94.3	1313.3	88	12	70	30
Case 3	07:46:44	6.6	310.7	130.0	4073.0	88	59	35	2420
Case 4	16:50:47	3.8	222.1	85.1	670.3	70	21	110	15
Case 5	11:32:11	4.7	254.3	104.4	1830.0	88	10	54	60

## 2 Experimental design, materials, and methods

### 2.1 Vessel and voyage

**Fig. 2:**
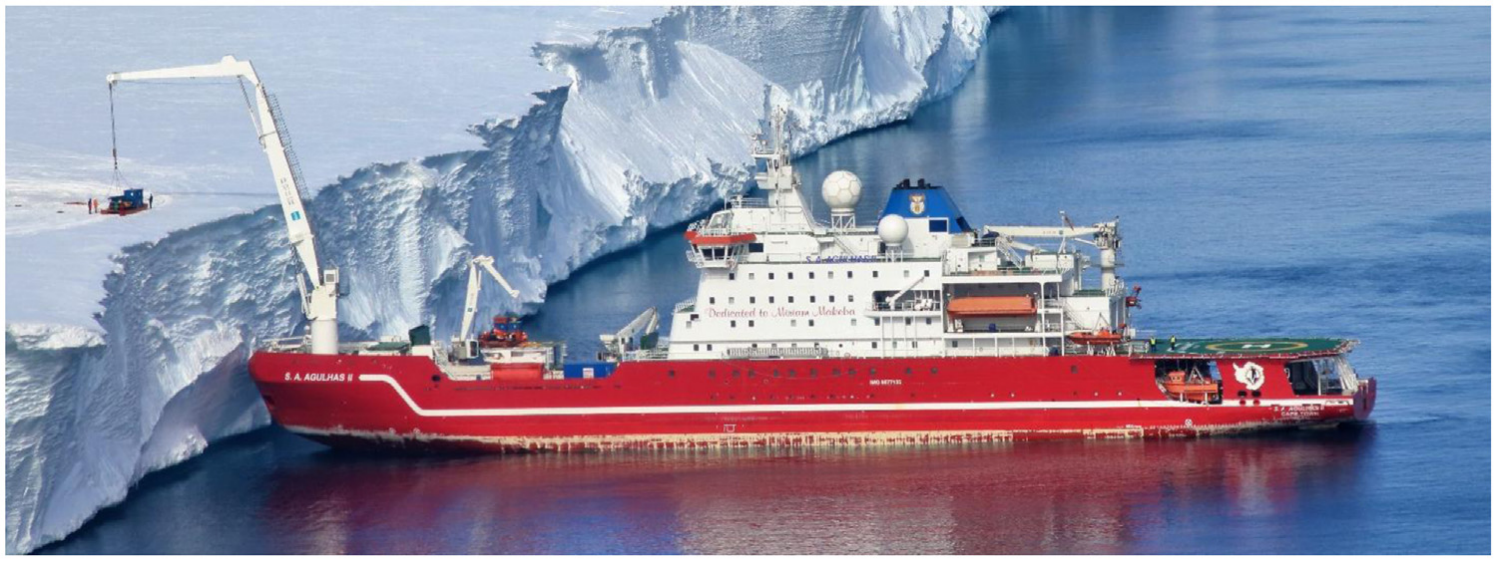
SAA II vessel was instrumented for the 2015/2016 relief voyage Antarctica.

The SAA II, depicted in Fig. 2, was manufactured in Rauma shipyard in 2012 by STX Finland [1]. Her hull is strengthened in accordance with DNV ICE-10 and the vessel classified to Polar Ice Class PC-5, which rates her capabilities for year-round operations in medium first-year ice containing old ice inclusions (International Association of Classification Societies, 2011). The ship is propelled by four 3 MW diesel generators which power two Conver Team electric motors of 4.5 MW each. She is equipped with two four-bladed variable pitch propellers with individual shaft lines [16]. The SAA II has open propellers and a direct diesel to electric drive to the propulsion shaft. Some additional specifications of the vessel are presented in Table 2.

**Table 2:** Vessel specifications of the SA Agulhas II [16].

**Gross tonnage**	12,897 t	**Main engine maker**	Wärtsilä
**Length / Breadth**	134 m / 22 m	**Diesel engine type**	6L32
**Classification**	Det Norske Veritas	**Electric motor type**	N3 HXC 1120 LL8
**Class notation**	1A1 PC-5/ICE-10	**Speed / Power at MCR**	140 rpm / 4500 kW
**Yard**	STX Finland, Rauma, Finland	**Nominal torque**	307 kNm
**Year built**	2012	**Propeller maker**	Rolls-Royce
		**No. of blades / Diameter**	4 / 4.3 m
		**Shaft characteristics**	Direct drive
		**No. of motors / propellers**	2 / 2

**Fig. 3:**
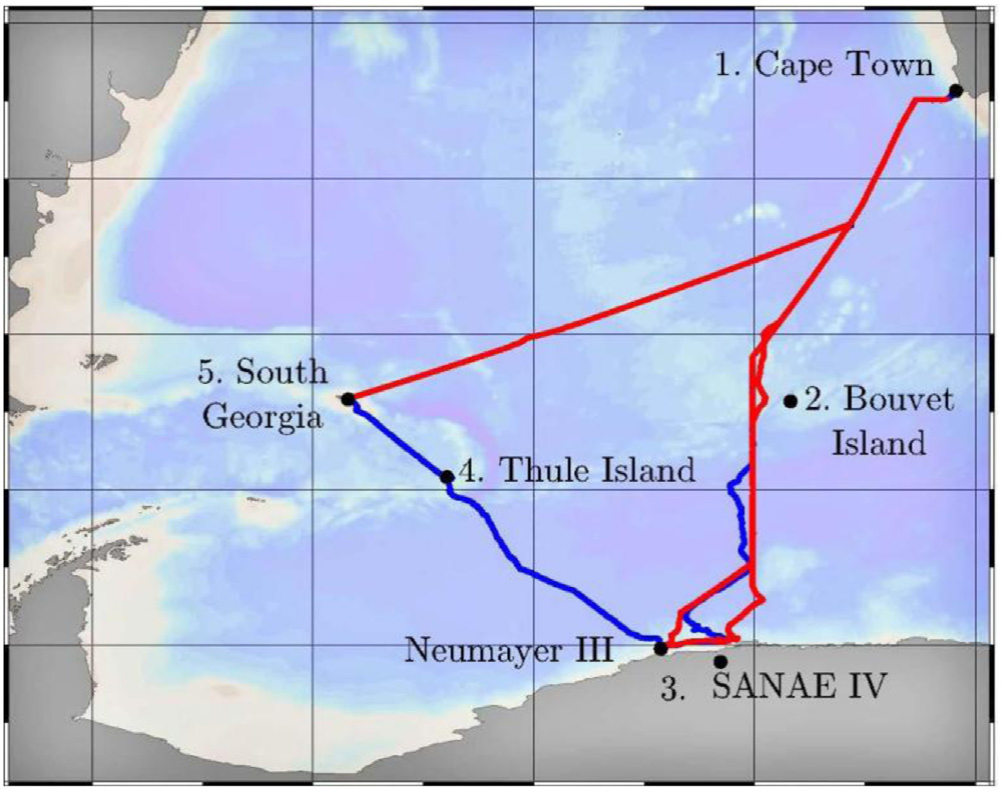
A GPS track of the 2015/2016 Antarctic relief voyage of the SAA II.

**Fig. 4:**
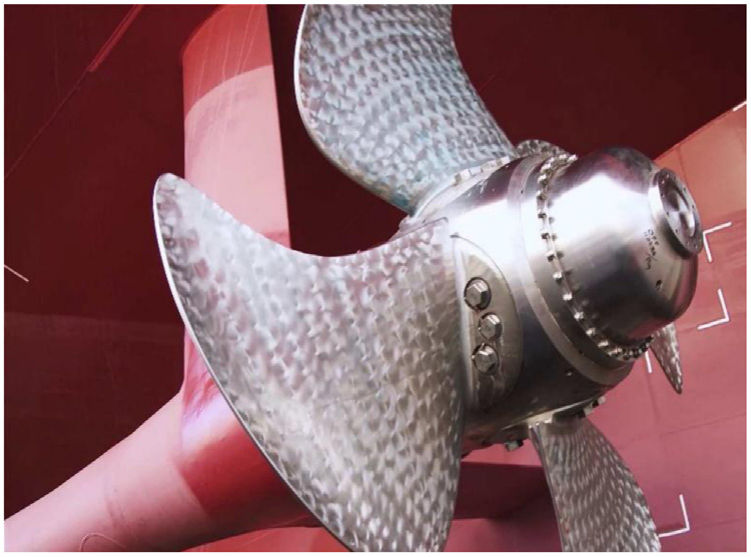
The four-bladed, variable-pitch propeller of the SAA II.

Shaft-line measurements were performed during the 2015/2016 Antarctic relief voyage of the SAA II as presented by the GPS track in Fig. 3. A photograph of the four-bladed propellers is shown in Fig. 4. The vessel departed from Cape Town Harbour (1) and headed south along the Greenwich Meridian. Ice was encountered prior to reaching the ice shelf at Penguin Bukta (3). From here the vessel departed for Akta Bukta near the German Antarctic Research Station, Neumayer, and continued through heavy pack ice towards the South Sandwich Islands, South Thule (4). She exited the ice field and reached South Georgia (5). She then returned to her original course from Cape Town and sailed south to Penguin Bukta (3) and SANAE IV before heading back to Cape Town (1).

### 2.2 Visual ice observations

Visual observations of ice conditions were performed from the bridge of the vessel when operating in ice as comprehensively documented by Suominen et al. [17]. The observations were executed in round-the-clock two- to three hour shifts with five to seven observers in the group to mitigate observer fatigue. Observations were reported in 10 min intervals and included ice thickness, ice concentration, snow thickness, brash ice amount, floe size as well as general comments. The thickness, concentration, and floe size were estimated as occurrence percentages (in tenths) for given categories during the measurement period.

**Fig. 5:**
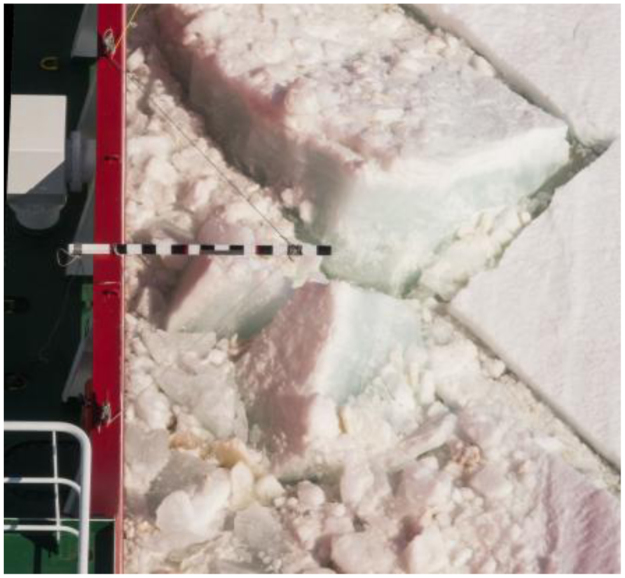
The measurement stick for ice thickness estimation. Each marker is 10 cm wide.

The ice thickness was estimated by comparing the thickness of the cross-section of upturning ice debris which was scaled with the aid of a yard stick. The yard stick was suspended overboard from the main deck and was marked with 10 cm wide black and white markings to calibrate the estimations of observers, see Fig. 5. Observers estimated the ice thickness using the stick as it is visible from the bridge. The real ice thickness was obtained by scaling the observations with a factor of 1.5 in order to correct for the parallax error. The factor was determined based on the distances to the sea surface and measurement stick from the bridge. Observers were required to perform the classification of ice thickness in categories with 20 cm increments between 0 m to 2 m with a final category for ice thickness in excess of 2 m (see Table 3). Additional thickness classes 2.0–2.5 m, 2.5–3.0 m and >3.0 m were added to observation classes for the 2015/2016 voyage. A limitation is that as the yard stick is only 1.5 m long, therefore the uncertainty increases significantly for the thickness classes exceeding 2.0 m. The average ice thickness for a 10 min period was determined by calculating a weighted average from the thickness observation periods.

**Table 3:** An extract from the subjective ice observation data template, which shows an example of ice concentration estimates [17]. A similar process was followed for the subjective assessment of floe size and ice thickness.

Time UTC +0									Snow [cm]		Ice concentration in tenths									
			Start		End						0–10	10–20	20–30	30–40-	40–50	50–60	60–70	70–80	80–90	90–100
Year	mm	dd	hh	mm	hh	mm	Lat	Lon	min	max										
2013	12	22	8	0	8	10	−70.46	−8.426		50	6			3	1					
2013	12	22	8	10	8	20	−70.45	−8.379		60	0				1	4		3	2	
2013	12	22	8	20	8	30	−70.45	−8.377			4							2	1	3
2013	12	22	8	30	8	40	−70.45	−8.376		60	0						3	4		3

The concentration of the ice field was estimated from inboard observations from conditions experienced in the close vicinity of the ship. As the crew preferably navigated in open water instead of ice, the ship followed open water leads in the ice whenever possible. In this case, the concentration was marked as zero, although floes of ice could be seen. The range from 0 to 100% was divided into categories with 10% increments, i.e. 0–10%, 10–20%, for observations of ice concentration. Here, zero denotes open water and 100% indicates complete ice cover. Table 3 presents an example from a part of the visual observation sheet.

Ice floes were categorized in terms of diameter in categories which included <20 m, 20–100 m, 100–500 m, 500–2000m, 2–5 km, >5 km. The classes were selected based on the egg code [2] used, for example, in the Baltic Sea. The floe diameters were estimated with the help of the main dimensions of the ship. If the floes were smaller than the width of the ship (~20 m), those belonged to the first class. If the floes were larger than the breadth, but smaller than the length of the ship (~120 m), those belonged to the class 20–100 m. If the floes were larger than the ship length, the diameter was estimated in multiples of the ship length.

**Fig. 6:**
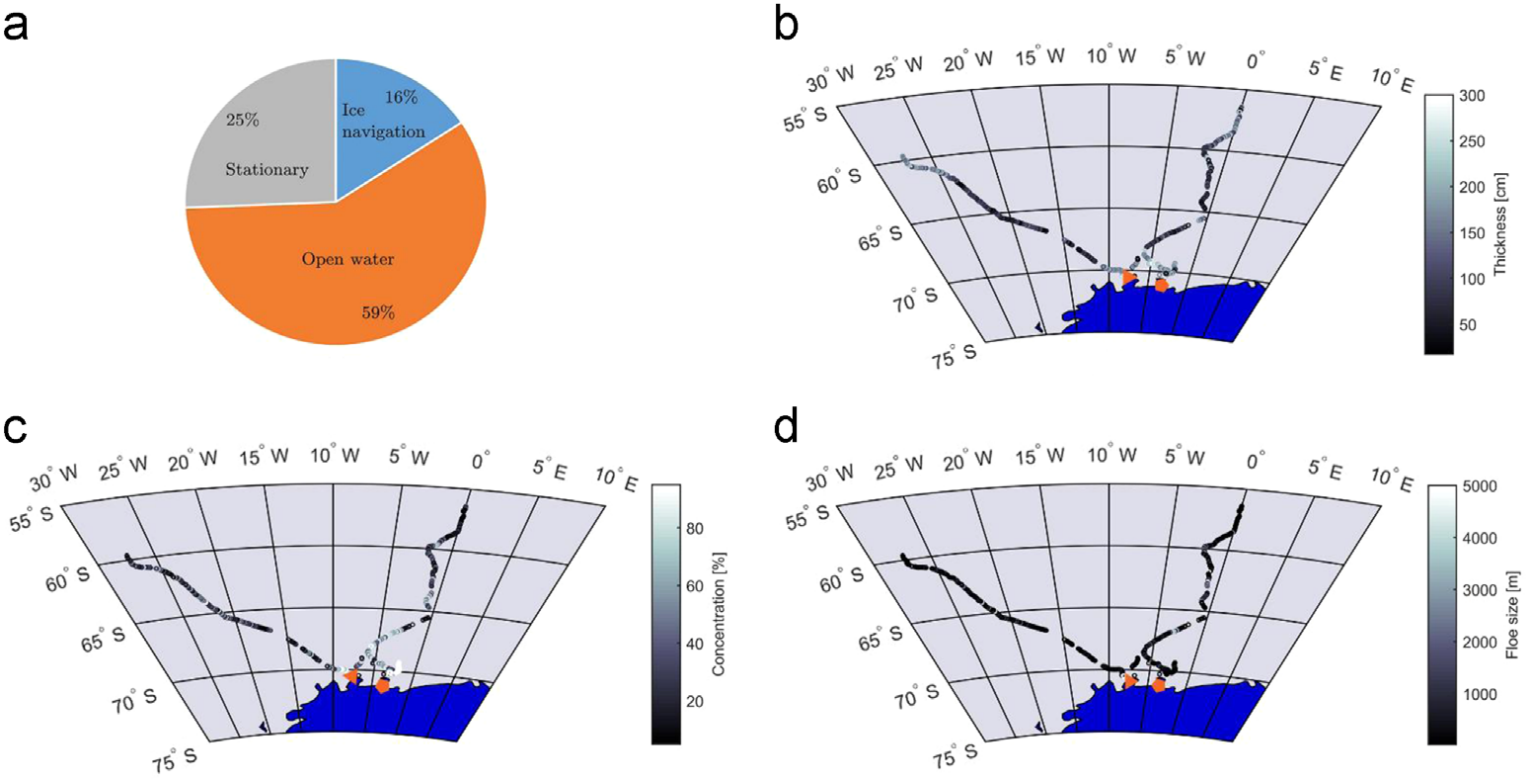
The (a) operational profile and ice conditions, including (b) thickness (c) concentration and (d) floe size encountered by the SAAII on the 2015/2016 Antarctic relief voyage.

The total voyage lasted 68 days, of which 10.7 days were spent navigating in ice, 40 days navigating in open water and 17.5 days stationary. The pie chart in Fig. 6a depicts the operational profile of the vessel. The ice conditions varied throughout the voyage and are summarized in Fig. 6b-d.

### 2.3 Full-scale measurements

**Fig. 7:**
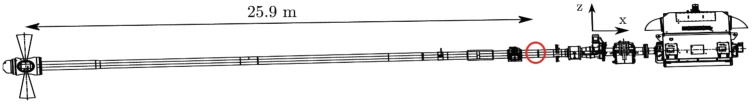
Location of strain gauges mounted along the shaft line.

**Fig. 8:**
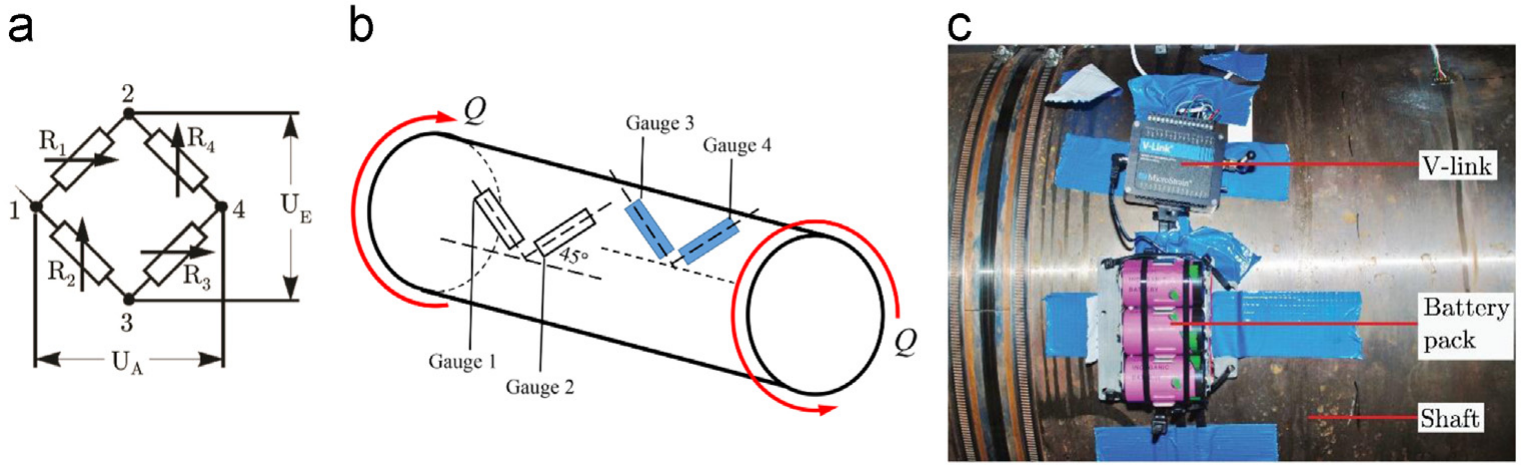
Shaft line measurements with (a) the Wheatstone bridge configuration (b) strain gauge placement and orientation and (c) measurement setup on the shaft-line of the SAA II.

.Strain gauges were installed on the port side intermediate shaft line, 25.9 m from the center of gravity of the propeller (Fig. 7), to determine torque loading from strain gauge measurements. The strain gauges were connected in a Wheatstone bridge configuration to reject axial strain, compensate for temperature variations and reject bending. This was achieved by installing two pairs of T-rosette strain gauges on diametrically opposing sides of the shaft. The gauges were inclined at ±45° with respect to the horizontal mid-plane of the shaft in order to measure the maximum shear stress on the outer surface (Fig. 8a). A V-link lossless extended range synchronized (LXRS) system produced by LORD MicroStrain, was installed to transmit the measurements wirelessly (Fig. 8b) to a HBM Quantum mobile data acquisition system. The HBM Quantum was connected to a laptop via an Ethernet cable and recorded through Catman AP V3.5 software at a sample rate of 600 Hz.

Fig. 8a provides a diagram of the Wheatstone bridge layout, indicating the supply voltage, $U_{E}$, and output voltage, $U_{A}$, as well as the strain gauge resistances ($R_{1}$ to $R_{4}$) for the four gauges in a full bridge. Fig. 8b depicts the orientation of the strain gauges for shear strain measurement on the shaft and Fig. 8c shows the physical installation. The bridge was set up to reject both axial- and bending strain whilst compensating for temperature variations. The gauge factor, k=1.99, is supplied on the packaging and $\varepsilon_{i}$, where (i=1,2,3,4) represent the strain measurements from gauges 1,2,3 and 4 of the Wheatstone bridge. When a torsional moment is applied, with the sense indicated in Fig. 8b, strain gauge 2 and 4 will sense a negative strain and strain gauge 1 and 3 will sense an equal and opposite positive strain. The resultant voltage, $U_{A}$, is obtained through the relationship:

(1) $$\frac{U_{A}}{U_{E}}=\frac{k}{4}\left(\varepsilon_{1}-\varepsilon_{2}+\varepsilon_{3}-\varepsilon_{4}\right)$$

The torque in the shaft, $Q_{\mathrm{shaft}},$ is determined from the output voltage of the Wheatstone bridge through:

(2) $$Q_{\mathrm{shaft}}=U_{A}\frac{\pi E(d_{0}^{4}-d_{i}^{4})}{16U_{E}kd_{0}\left(1+\nu \right)}$$

Here, E, is the Young׳s modulus, ν, is the Poisson׳s ratio and $d_{0}$ and $d_{i}$ respectively reflect the outer and inner diameters of the hollow shaft. The strain gauge factor, k, is directly obtained from manufacturer specifications. The shaft dimensions for the SAA II were obtained from engineering drawings by STX Finland [16]. The material specifications were sourced from Rolls-Royce [15], which provided parameters for numerical calculations during the propulsion system design phase. The dimensions, material properties and shaft related variables are presented in Table 4. The depth of the propeller centerline, $h_{0}$, was not directly obtainable from engineering drawings and was inferred from scaled vessel drawings.

**Table 4:** Shaft line dimensions, material properties and shaft related variables at the measurement locations [3], [6], [14], [15], [16].

Description	Symbol	Value	Description	Symbol	Value
Modulus of elasticity	*E*	210 GPa	Max ice thickness	*H*_*ice*_	2.0 m
Shear modulus	*G*	81 GPa	Ice strength index	*S*_*ice*_	1.1 m
Outer diameter	$d_{0}$	0.5 m	Pitch at 70% of blade radius	*P*_*0.7*_	5.15 m
Inner diameter	$d_{i}$	0.175 m	Expanded blade area ratio	*EAR*	0.51
Hub diameter	$d_{h}$	1.32 m	Depth of propeller centerline	$h_{0}$	3.75 m

### 2.4 Inverse methods

Ice-induced moments on the propeller are to be determined from indirect shaft line measurements. This is achieved through a two-step process. Firstly, a forward problem is solved whereby the dynamic model is subjected to a step impulse moment at the propeller. The impulse response function between the externally applied ice moment on the propeller and the internal torque response in the shaft line is thereby determined at the measurement location. Secondly, an inverse problem is solved to determine externally applied propeller moments from the measured shaft line torque and ill-posed inverted impulse response.

A simplified dynamic model of the torsional dynamic response of the SAA II was obtained by using a lumped mass model documented by Rolls-Royce [15] and Ikonen et al. [11]. This was done to determine the impulse response function, which describes the strain gauge output when a unit moment is applied, at t=0, to the propeller. The governing matrix equation for the torsional response of a mass-damper system is:

(3) $$J\ddot{\bar{\theta }}(t)+C\dot{\bar{\theta }}(t)+K\bar{\theta }(t)=\bar{Q}(t)$$

Here, J, is a matrix containing entries, which relate to the polar moment of inertia, C, the damping, K, the stiffness, $\bar{Q}$, the generalized excitation torque vector and $\bar{\theta }$, the angular displacement vector of the twisting angles at the system nodes.

**Fig. 9:**
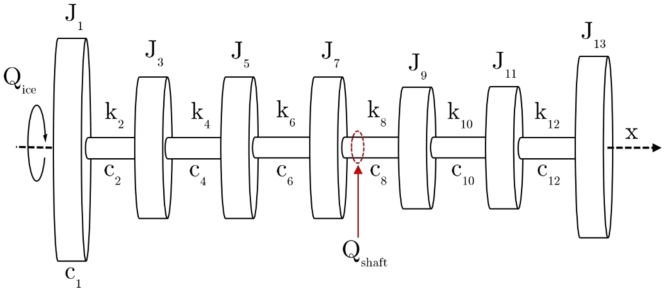
A diagram of the dynamic model for the SAA II shaft line comprising inertia, damping and torsional spring elements [15].

A diagram of the lumped-mass model for the SAA II shaft line is shown in Fig. 9. $J_{1}$ represents the controllable pitch propeller (CPP), $J_{3}$ the mid-propeller shaft, $J_{5}$ the sleeve coupling, $J_{7}$ the oil distribution box flange, $J_{9}$ the thrust shaft collar, $J_{11}$ the electric motor flange and $J_{13}$ the propulsion motor. The hydro-dynamic damping on the rotating propeller is modelled by $c_{1}$ whereas $c_{2}$*,* $c_{4}$*, …,* $c_{12}$ and $k_{2}$*,* $k_{4}$*, …,* $k_{12}$ respectively represent the shaft line damping and torsional stiffness. ${\bar{Q}}_{\mathrm{shaft}}$ is the shaft torque vector, which is calculated from full-scale measurements using Eq. 2. Inverse methods are subsequently required to determine the ice-induced moment at the propeller, ${\bar{Q}}_{\mathrm{shaft}}$. The variables used for the parameters of the dynamic model were obtained from Rolls-Royce documentation [30] as presented in Table 5.

**Table 5:** Parameters for the lumped-mass model as published by Rolls-Royce [15].

Variable	Description	Value
*J*_1_	Propeller	1.347 × 10^4^ kg m^2^
*J*_3_	Mid propeller shaft	5.590 × 10^2^ kg m^2^
*J*_5_	Sleeve coupling	5.120 × 10^2^ kg m^2^
*J*_7_	OD box flange	4.870 × 10^2^ kg m^2^
*J*_9_	Thrust shaft collar	1.410 × 10^2^ kg m^2^
*J*_11_	Motor flange	1.740 × 10^2^ kg m^2^
*J*_13_	Propulsion motor	4.415 × 10^3^ kg m^2^
*c*_1_	Water damping	1.136 × 10^5^ Nm s*/*rad
*c*_2*,*4*,.,*12_	Steel shaft	1.800 × 10^2^ Nm s*/*rad
*k*_2_	Steel shaft	5.950 × 10^7^ Nm rad
*k*_4_	Steel shaft	5.950 × 10^7^ Nm rad
*k*_6_	Steel shaft	1.120 × 10^8^ Nm rad
*k*_8_	Steel shaft	6.930 × 10^8^ Nm rad
*k*_10_	Steel shaft	5.090 × 10^8^ Nm rad
*k*_12_	Steel shaft	1.430 × 10^8^ Nm rad

**Fig. 10:**
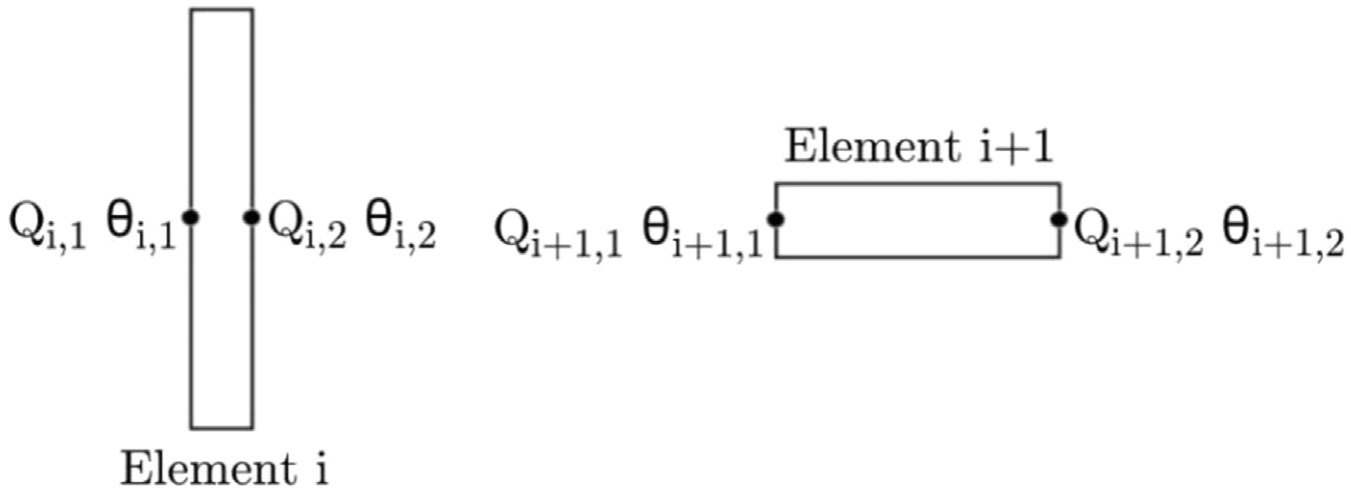
A diagram depicting the elements and relative nodes of the SAA II shaft-line model.( Adapted from [11]).

In order to determine the ice-induced torque at the propeller, rotational degrees of freedom were defined along the longitudinal shaft axis (x-axis). The model comprised two types of elements, namely inertia elements and shaft elements. The respective elements each contained two nodes as shown in Fig. 10. Each node is associated with a torsional moment, Q, and angular displacement, θ. Using the governing equation of torsional vibration in Eq. (3), Eq. (4) is derived for inertia elements (corresponding to odd values of i) and Eq. 5 for torsional spring elements (even values of i):

(4) $$J_{i}{\ddot{\theta }}_{i,1}+c_{i}{\dot{\theta }}_{i,1}=-Q_{i,1}+Q_{i,2}+Q_{\mathrm{ice}}$$

(5) $$c_{i}\left({\dot{\theta }}_{i,2}-{\dot{\theta }}_{i,1}\right)+k_{i}\left(\theta_{i,2}-\theta_{i,1}\right)=Q_{i,1}$$

with i being the increment for the thirteen elements of the shaft line system. Subscript (i,1) denotes the value of the variable on the left side of the element and subscript (i,2) on the right side of the element. For inertia elements, the angular displacement on the right and left sides are equal, and therefore, $\theta_{i,1}=\theta_{i,2}$. For spring elements, the internal torque remains constant and therefore, $Q_{i,1}=Q_{i,2}$.

Direct integration methods could be used to obtain approximate solutions of dynamic systems [20]. Two principal approaches to multi-degree of freedom direct integration methods include explicit and implicit schemes [4]. For an explicit scheme, previously determined values of displacement, velocity and acceleration are used to determine the response quantities. Implicit schemes combine the equations of motion with difference equations to calculate the displacement directly. Implicit schemes involve iterative procedures for each time step, making them more computationally intensive [20]. The disadvantage of explicit schemes is that they are conditionally stable relative to the size of the selected time step, whereas implicit schemes can be either conditionally or unconditionally stable. Wilson [20] recommends that single-step, implicit, unconditionally stable methods should be used for step-by-step analysis of practical structures. To this end, the unconditionally stable Newmark-Beta method was used for direct integration in the time domain as outlined by Ikonen et al. [11].

The dynamic model was solved by first defining an integration formula for the angular velocity, $\dot{\theta }$, and angular acceleration, $\ddot{\theta }$, through the Newmark-Beta method [20]. The Average Acceleration Method is used for the Newmark-Beta integration with parameter values α=0.5 and β=0.25. Wilson [20] recommends that these parameter values will result in no energy dissipation with good accuracy for small time steps. The dynamic problem is solved by combining the Newmark-Beta integration method using an incremental form of the governing equation of torsional vibration. For a more detailed procedure, the reader is encouraged to refer to De Waal [18] and Ikonen et al. [11]. (Ikonen et al., All calculations were performed using custom algorithms programmed in MATLAB.

#### 2.4.1 Inverse methods

The principle of superposition [12] is used to model the response of linearly elastic dynamic systems. The relationship between the shaft torque and ice-induced moment is expressed by the convolution integral in Eq. (6), which represents the dependency between the loading on the propeller, $Q_{\mathrm{ice}}$, and the response measured on the shaft line, $Q_{\mathrm{sha}\mathrm{ft}}$. H is the impulse response function between the loading point at the propeller and the measurement location on the shaft. The impulse response function is shifted by the variable of integration φ to represent a random load history as a series of impulses [12].

(6) $$Q_{\mathrm{shaft}}(t)=\int_{0}^{t}H(t-\varphi )Q_{\mathrm{ice}}(\varphi )d\varphi$$

Eq. 6 can be solved by transforming it into a system of linear equations and discretizing the integral into time steps, which results in Eq. (7) [13]:

(7) $${\bar{Q}}_{\mathrm{shaft}}(t)=H(t){\bar{Q}}_{\mathrm{ice}}(t)$$

Here, H is the impulse response matrix representing the transfer function between the loading point at the propeller and the measurement location on the shaft, and ${\bar{Q}}_{\mathrm{shaft}}$ and ${\bar{Q}}_{\mathrm{ice}}$ respectively represent the shaft- and ice-induced moment vectors. In order to solve for the unknown ice-induced moment vector, ${\bar{Q}}_{\mathrm{ice}}$, from shaft line measurements, ${\bar{Q}}_{\mathrm{shaft}}$, Eq. (7) is rearranged, as presented in Eq. (8). This results in the requirement to solve an inverse problem in order to determine the causal factors that produce the observed response.

(8) $${\bar{Q}}_{\mathrm{ice}}(t)=H^{-1}\left(t\right){\bar{Q}}_{\mathrm{shaft}}(t)$$

The complication with the discretization of inverse problems is that this leads to an ill-conditioned coefficient matrix for the system of linear equations, which require regularization methods to obtain stable solutions [7]. Regularization is the procedure whereby the initial problem is modified to reduce the sensitivity of the response towards a robust solution [13].

To this end three inverse methods have been investigated to perform inverse moment determination in an impact loading situation of the dynamic shaft line structure. In keeping with the approach of Ikonen et al. [11] three regularization methods, namely Truncated Singular Value Decomposition (TSVD), Truncated Generalized Singular Value Decomposition (TGSVD) and Tikhonov regularization were implemented.

TSVD is a common method used to regularize ill-posed systems. The SVD of $H\in R^{m\times n}$, where m≥n, can be defined as [9]:

(9) $$H=U\Sigma V^{T}=\sum_{i=1}^{n}{\bar{u}}_{i}\sigma_{i}{{\bar{v}}_{i}}^{T}$$

Here, U is a matrix of orthonormalized eigenvectors of $HH^{T}$ and V comprises the orthonormalized eigenvectors of $H^{T}H$. Furthermore, Σ is a diagonal matrix containing non-negative singular values of H in decreasing order. As expressed in Eq. (9) the solution of the system depends on the singular values, $\sigma_{i}$, and singular vectors (${\bar{u}}_{i}$ and ${\bar{v}}_{i}$) of H. TSVD aims to reduce the rank of the matrix, H, by eliminating small singular values, thereby obtaining a closest well-conditioned approximation. This is achieved by evaluating the magnitude of the singular values. If a discontinuity occurs where the singular values decrease rapidly in magnitude, the larger singular values are retained and the remainder are set equal to zero [10]. Eq. (9) can be rewritten to obtain the desired solution through the TSVD method, where, j, represents the number of singular values retained [10] and ${\bar{q}}_{s}$ is the internal shaft torque:

(10) $${\bar{Q}}_{\mathrm{ice}}=\sum_{i=1}^{j}\frac{{{\bar{u}}_{i}}^{T}{\bar{q}}_{s}}{\sigma_{i}}{\bar{v}}_{i},\;j\leq n$$

Truncated Generalized Singular Value Decomposition (TGSVD) is a more sophisticated method whereby further information about the desired solution can be incorporated to stabilize the problem [13]. This is achieved through the regularization matrix, L, which often takes the form of the first or second derivative operator [7]. Ikonen et al. [11] found that the first order regularization matrix (Eq. (11)) is well-suited to smooth the obtained propeller moment solution. It should be noted that, since the elements of the solution correspond to changes in the ice-induced moment vector, $\bar{M}={[∆m_{1}∆m_{2}∆m_{3}\ldots ∆m_{n}]}^{T}$ the regularization in fact corresponds to smoothing the solution by the second order derivative.

(11) $$L=\left[\begin{array}{ccccc} -1 & 1 & 0 & \ldots & 0\\ 0 & -1 & 1 & \ldots & 0\\ \vdots & \vdots & \ddots & \ddots & \vdots \\ 0 & 0 & \ldots & -1 & 1 \end{array}\right]$$

The system can be represented by the real matrix pair ($H\in R^{m\times n}$ and $L\in R^{p\times n}$) with m≥n≥p [5]:

(12) $$H=U\left[\begin{array}{cc} \Sigma & 0\\ 0 & I_{n-p} \end{array}\right]X^{-1}$$

(13) $$L=V(\bar{M},0)X^{-1}$$

Here, $U\in R^{p\times r}$ and $V\in R^{q\times q}$, which have orthonormal columns. Therefore, $UU^{T}=I_{r}$ and $V^{T}V=I_{q}$. Furthermore, $X\in R^{r\times r}$ is a non-singular matrix. The desired solution can be obtained by applying TGSVD, which is similar to TSVD wherein the number of singular values is reduced to j [10]:

(14) $${\bar{Q}}_{\mathrm{ice}}=\sum_{i=p-j+1}^{p}\frac{{{\bar{u}}_{i}}^{T}{\bar{q}}_{s}}{\sigma_{i}}x_{i}+\sum_{i=p+1}^{n}{({{\bar{u}}_{i}}^{T}{\bar{q}}_{s})x}_{i}$$

Another widely used regularization method is Tikhonov׳s regularization method, which involves the solution of a least squares problem. This method is convenient for problems in which both the coefficient matrix and the required solution can only be determined approximately [7]. This method filters out the unwanted components corresponding to small singular values by adding damping to each TSVD component of the solution. The formulation of Tikhonov׳s method is [7]:

(15) $$\min\left\{{\left\|H{\bar{Q}}_{\mathrm{ice}}-{\bar{Q}}_{\mathrm{shaft}}\right\|}_{2}^{2}+\lambda {\left\|L{\bar{Q}}_{\mathrm{ice}}\right\|}_{2}^{2}\right\}$$

Here, *λ* is a positive constant referred to as the regularization parameter. The required solution for the ice moment vector, ${\bar{Q}}_{\mathrm{ice}}$, minimizes the jectivi function for Thikonov׳s method in Eq. (15).

#### 2.4.2 Validation and determination of regularization parameters

The solution of ill-posed inverse problems using TSVD and the Tikhonov methods, require the determination of the respective regularization parameters, $n_{e}$ and λ. To validate that the applied regularization methods were implemented correctly, a known ice moment impulse was applied as described by Ikonen et al. [11]. This has three purposes: firstly, the feasibility of the method is evaluated; secondly, the optimum levels of regularization for the application of real data can be determined and thirdly, the different methods can be compared to one another to determine their relative strengths.

**Fig. 11:**
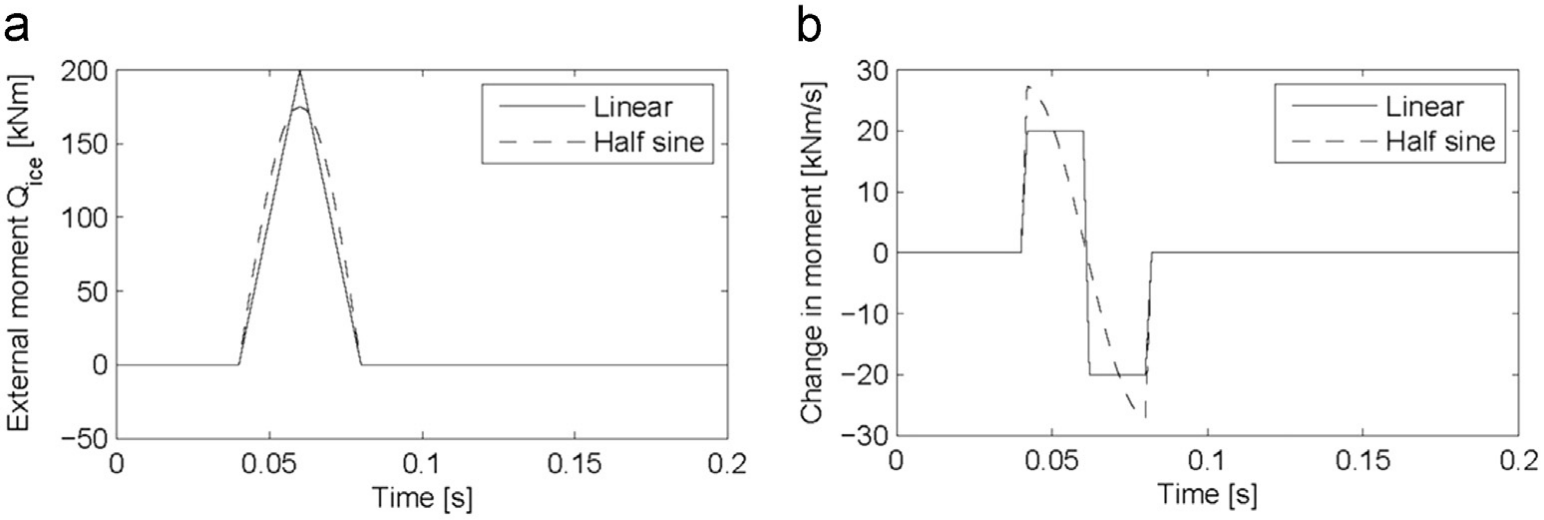
Synthesized data for linear and half sine moment impulses of 40 ms duration presented as (a) a function of time and (b) the first derivative as a function of time.

Synthesized moment impulses were created, with the requirement that it be representative of a real ice induced loading moment. A linear impulse of 40ms duration and a peak of 200kNm was used, as well as a half sine impulse also of 40ms duration and a maximum value of 175kNm. These impulses therefore represent potential ice impacts with sharp and round peaks. The duration of the impulse is based on the modelled torque excitation for a 90 degree single blade impact sequence of a four-bladed propeller. Furthermore, the impulse duration was also selected for algorithm validation by Ikonen et al. [11] and selection of similar loadings would enable a comparison of the results obtained. These impulses are presented in Fig. 11a.

The dynamic model applies the change in external moment, therefore the first time derivative of the known moments need to be determined. This was done with a time step of 2ms, the equivalent to a sample frequency of 500 Hz, as presented in Fig. 11b. The change in external known moment is applied as in ice-induced moment. The hypothetical shaft-line response is then “recorded” at the model element corresponding to the measurement location on the shaft line. In order to avoid inverse crime, which is when the same, or very similar, theoretical information is employed to synthesize and invert data in an inverse problem [21], Ikonen et al. [11] suggested adding deviations to the verification data. Two types of deviations were added. Firstly, ±10% deviations were added to the inertia and torsional spring stiffness, which resembles the uncertainty of the dynamic model. Secondly, random deviations of ±650Nm were added to each data point of the verification data to model the uncertainty of the strain gauge measurements. This value corresponds to ±1% of the peak torque value measured on the propulsion shaft during ice-induced loading.

In order to apply inverse methods, the regularization parameters, $n_{e}$ and λ required determination. The L-curve was plotted, whereby the semi-norm is depicted against the residual norm. The optimal regularization values are located at the corner of the curve. If too much regularization is applied, then the solution will not fit the desired curve properly and if too little regularization is applied then the solution will fit the desired curve well but will be dominated by the contribution from the data errors [9]. The L-curve is used to find the best compromise between the two quantities that need to be controlled.

**Fig. 12:**
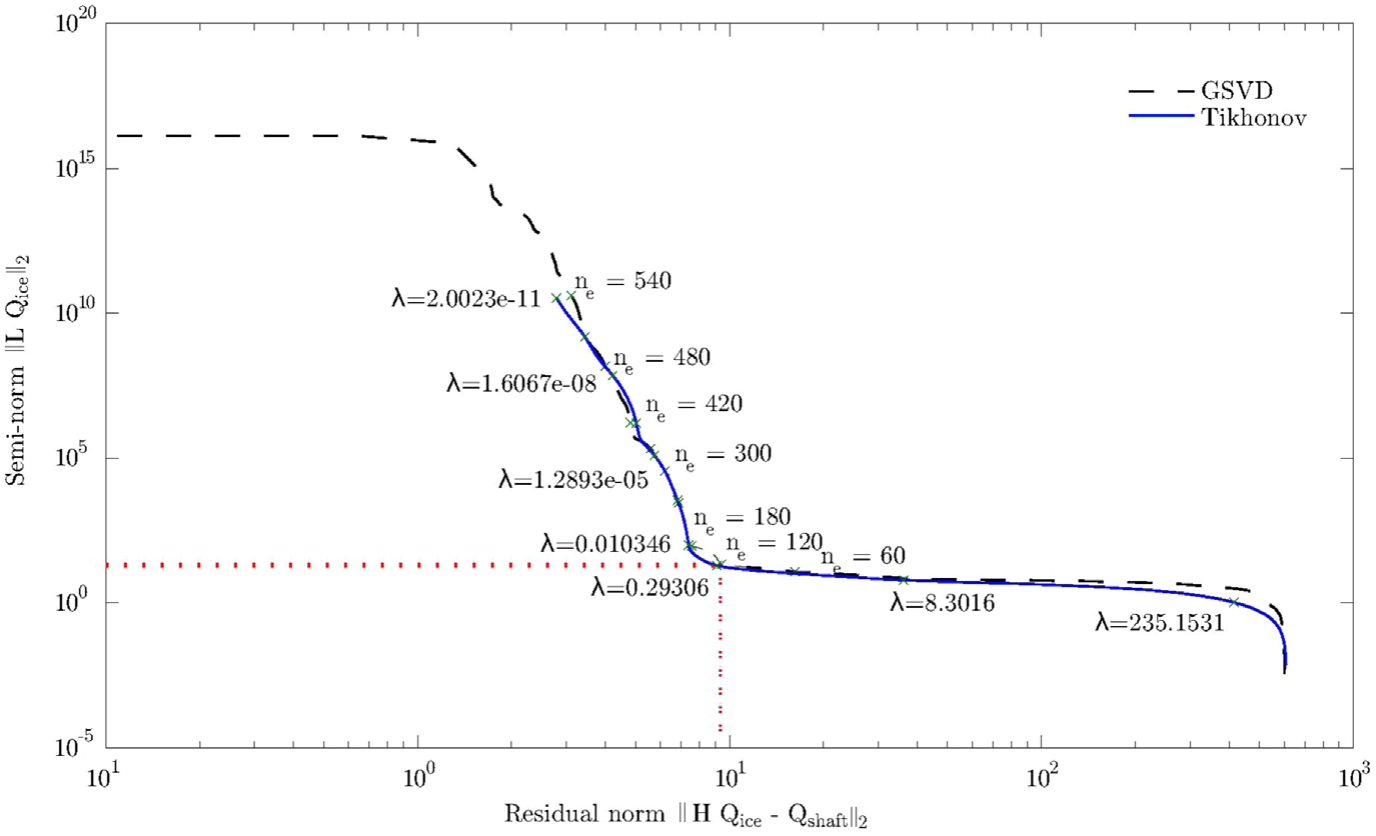
Comparison of TGSVD and Tikhonov L-curves for a synthesized linear moment impulse.

This plot is only applicable to the TGSVD and Tikhonov methods as SVD does not implement the L-matrix. The zero, first and second order regularization matrix L was evaluated for the current model and it was determined that the first order regularization matrix provided the best results for all three inverse methods. The optimum number of non-zero eliminated singular values for GSVD was determined to be $n_{e}=120$ and the optimum regularization parameter for the Tikhonov was determined to be $\lambda =24.57\times 10^{-2}.$ Compact truncated methods were used whereby only the non-zero eigenvalues with the corresponding eigenvectors were retained. These regularization methods were implemented using algorithms written by Hansen [8]. The L-curve for the linear moment impulse is presented in Fig. 12. The half sine moment impulse provided similar results.

#### 2.4.3 Algorithm

**Fig. 13:**
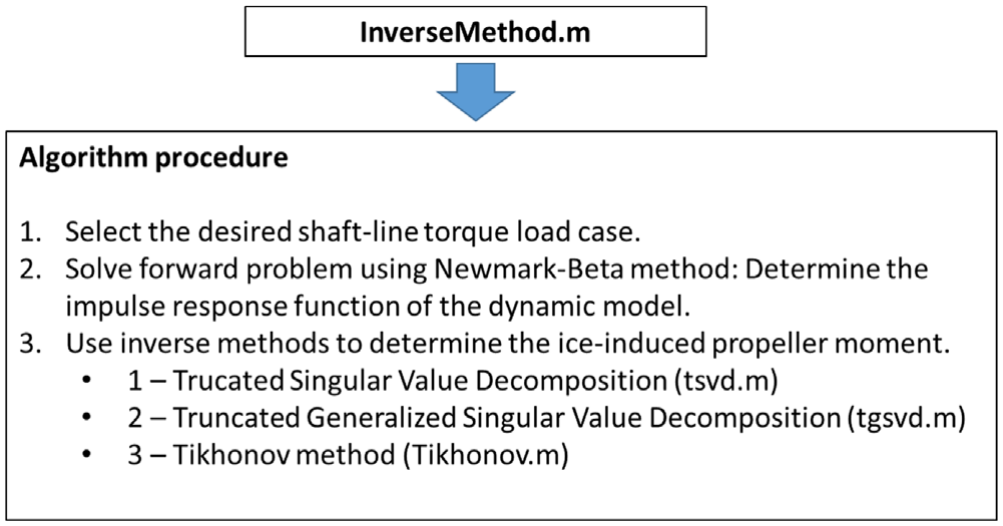
The calculation procedure used in InverseMethod.m.

Matlab algorithms are published with this data to load the load case data and perform the required inverse calculations. The procedure followed by the Matlab algorithm, InverseMethod.m is outlined in Fig. 13 and further highlighted in the fully commented algorithms attached to this submission.
