# Supplementary material for: Data for indirect load case estimation of ice-induced moments from shaft line torque measurements
Source: Data Brief. 2018 May 28;19:1222–36. doi: 10.1016/j.dib.2018.05.115 (PMC6140293; doi:10.1016/j.dib.2018.05.115)
Supplement: Supplementary file 1 — Supplementary material [file mmc1.pdf]

Conflict of Interest and Authorship Conformation Form

Please check the following as appropriate:

- All authors have participated in (a) conception and design, or analysis and interpretation of the data; (b) drafting the article or revising it critically for important intellectual content; and (c) approval of the final version.

- This manuscript has not been submitted to, nor is under review at, another journal or other publishing venue.

- The authors have no affiliation with any organization with a direct or indirect financial interest in the subject matter discussed in the manuscript

- The following authors have affiliations with organizations with direct or indirect financial interest in the subject matter discussed in the manuscript:

Author's name	Affiliation
Anriëtte Bekker	Stellenbosch University
Rosca J.O. De Waal	Stellenbosch University
Philippus S. Heyns	University of Pretoria
