# Supplementary figures and images for: Data for indirect load case estimation of ice-induced moments from shaft line torque measurements
Source: Data Brief. 2018 May 28;19:1222–36. doi: 10.1016/j.dib.2018.05.115 (PMC6140293; doi:10.1016/j.dib.2018.05.115)

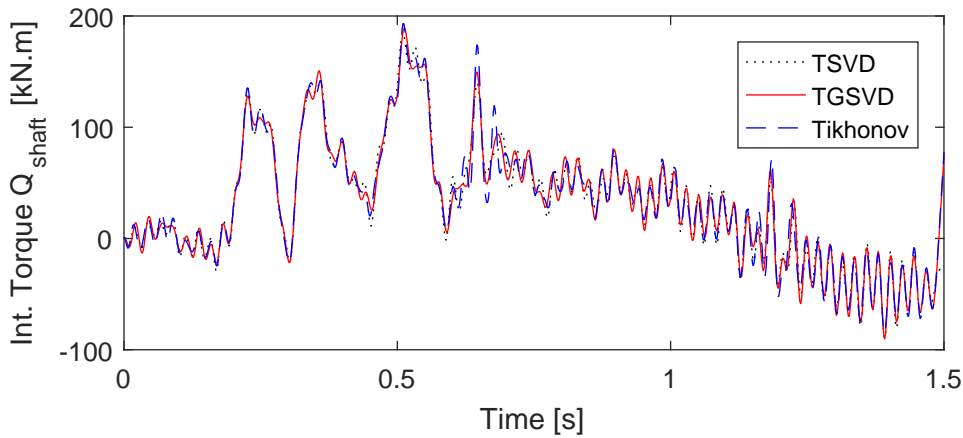

Supplement: Supplementary file 3 — Supplementary material [file mmc3.zip › Data/Case 1/Case1_IceTorque.pdf]

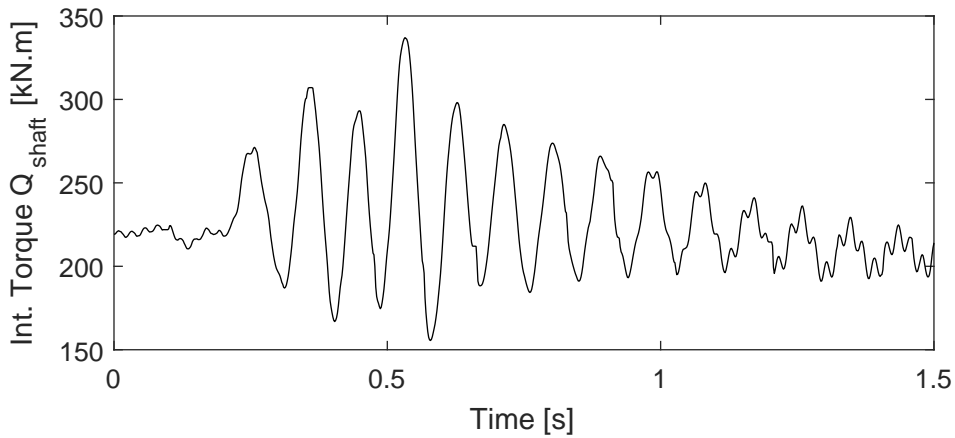

Supplement: Supplementary file 3 — Supplementary material [file mmc3.zip › Data/Case 1/Case1_ShaftTorque.pdf]

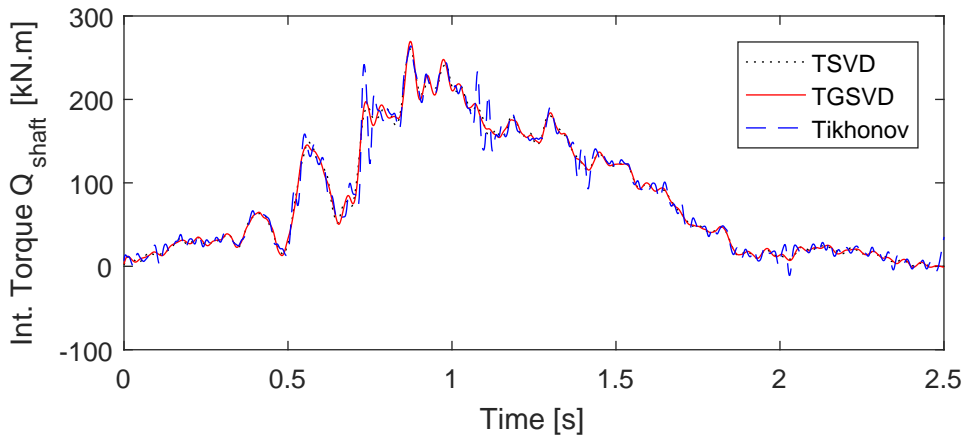

Supplement: Supplementary file 3 — Supplementary material [file mmc3.zip › Data/Case 2/Case2_IceTorque.pdf]

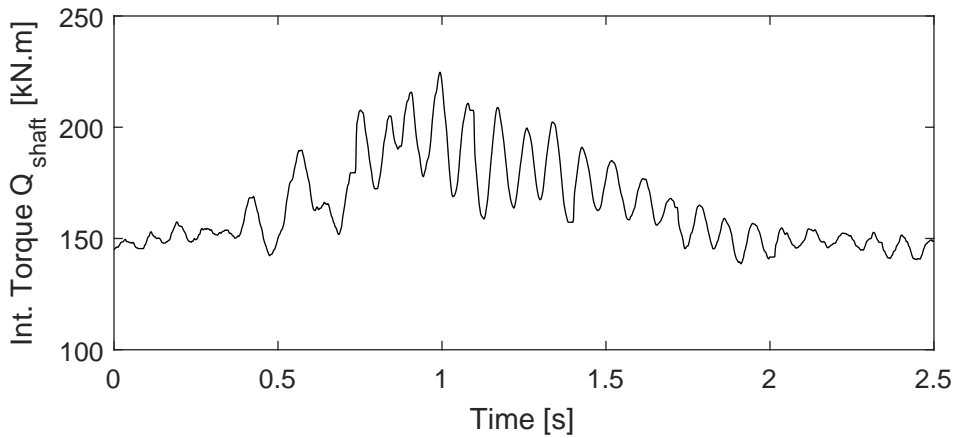

Supplement: Supplementary file 3 — Supplementary material [file mmc3.zip › Data/Case 2/Case2_ShaftTorque.pdf]

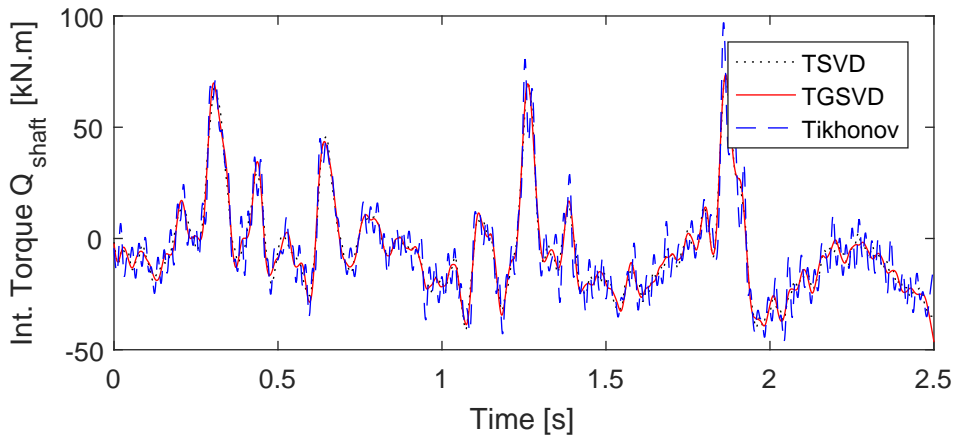

Supplement: Supplementary file 3 — Supplementary material [file mmc3.zip › Data/Case 3/Case3_IceTorque.pdf]

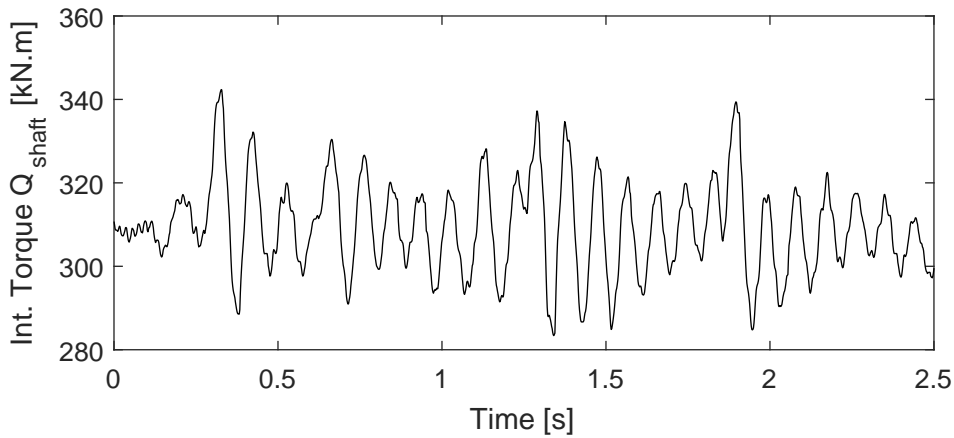

Supplement: Supplementary file 3 — Supplementary material [file mmc3.zip › Data/Case 3/Case3_ShaftTorque.pdf]

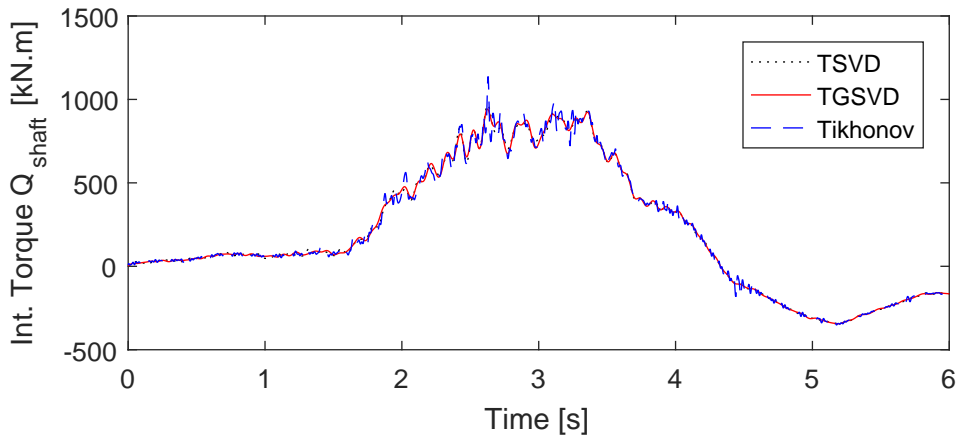

Supplement: Supplementary file 3 — Supplementary material [file mmc3.zip › Data/Case 4/Case4_IceTorque.pdf]

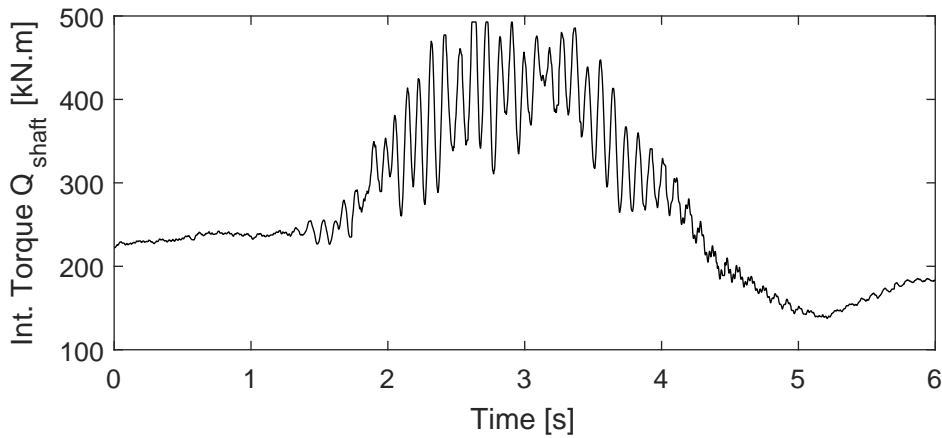

Supplement: Supplementary file 3 — Supplementary material [file mmc3.zip › Data/Case 4/Case4_ShaftTorque.pdf]

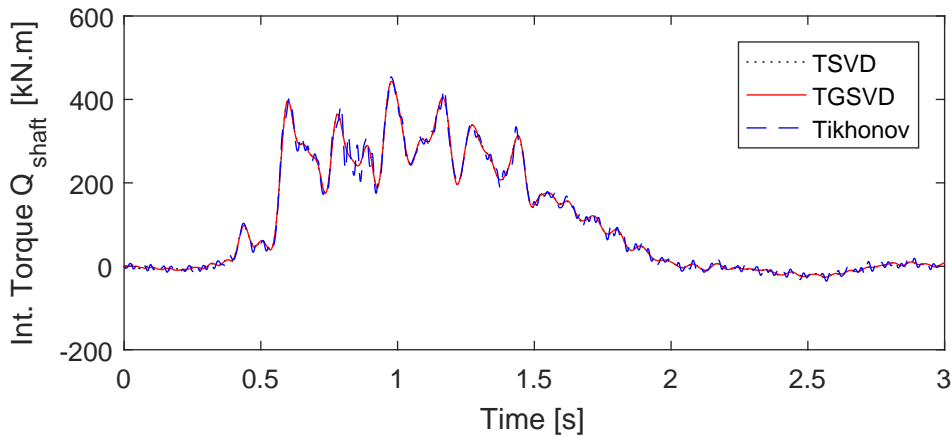

Supplement: Supplementary file 3 — Supplementary material [file mmc3.zip › Data/Case 5/Case5_IceTorque.pdf]

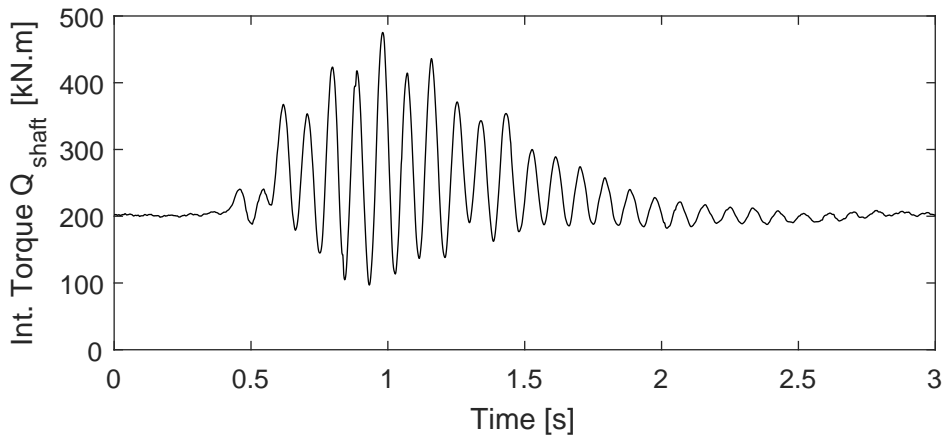

Supplement: Supplementary file 3 — Supplementary material [file mmc3.zip › Data/Case 5/Case5_ShaftTorque.pdf]
